# Detection of parasitic particles in domestic and urban wastewaters and assessment of removal efficiency of treatment plants in Tehran, Iran

**DOI:** 10.1186/s40201-015-0155-5

**Published:** 2015-01-25

**Authors:** Kareem Hatam-Nahavandi, Amir Hossein Mahvi, Mehdi Mohebali, Hossein Keshavarz, Iraj Mobedi, Mostafa Rezaeian

**Affiliations:** Department of Medical Parasitology and Mycology, School of Public Health, Tehran University of Medical Sciences, Tehran, Iran; Department of Environmental Health Engineering, School of Public Health, and Center for Water Quality Research, Institute for Environmental Research, Tehran University of Medical Sciences, Tehran, Iran; Center for Research of Endemic Parasites of Iran (CREPI), Tehran University of Medical Sciences, Tehran, Iran

**Keywords:** Protozoan (oo)cyst, Helminth egg, Wastewater treatment plant, Slaughterhouse, Iran

## Abstract

**Background:**

In recent years, decreasing annual rainfalls in some countries and population growth have led to a shortage of freshwater resources. Thus, recycled wastewaters has been suggested for agricultural activities. Contamination of wastewaters with pathogens is a major concern for the use of these waters. This study aimed to (i) investigate the occurrence of helminth eggs and protozoan (oo)cysts in human and livestock wastewaters, and (ii) evaluate the parasite removal efficiencies of urban and slaughterhouse treatment plants in Tehran province, Iran. One hundred and eight samples were collected from five urban and domestic wastewater treatment plants in Iran. Wastewater samples were concentrated by centrifugal-concentration and filtration methods.

**Results:**

The quantity of helminths egg and protozoa (oo)cyst per liter of urban raw wastewater ranged from 1.2 × 10^1^ to 2.9 × 10^1^ and from 9.6 × 10^2^ to 1.9 × 10^3^, respectively. The number of eggs and (oo)cysts per liter of animal raw wastewater ranged from 1.6 × 10^3^ to 4.9 × 10^3^ and 3.1 × 10^4^ to 6.0 × 10^4^, respectively. The helminths and protozoa identified in urban treatment plants included hookworms, *Hymenolepis* and *Rhabditis* (or probably *Strongyloides*), *Entamoeba*, *Isospora*, *Giardia*, *Chilomastix* and *Cryptosporidium*, while in slaughterhouses *Trichuris*, *Trichostrongylus*, *Moniezia*, *Dicrocoelium*, *Fasciola*, *Entamoeba*, *Cryptosporidium*, *Eimeria* and *Giardia* were isolated. The overall removal efficiency of eggs and (oo)cysts in the treatment plants ranged from 94.8 to 95.7% and from 79.3 to 85.8%, respectively.

**Conclusion:**

The study results revealed that the efficacy of removal of nematode eggs, and not protozoan (oo)cysts, in urban wastewater treatment plants, is in compliance with the WHO parasitological guideline (<1 nematode per liter) required for unrestricted irrigation.

## Background

Water is life. In 2025, water shortages will be more prevalent among poorer regions such as Africa and parts of Asia, where resources are limited, anuall rainfall is low, population growth is rapid, and water concumption is high [1]. Under these circumstances, treated wastewaters can be used for irrigation of parks, playgrounds and agricultural farmlands [2]. Contamination of wastewaters with pathogens and chemical pollutants is a major concern for the use of these waters. Parasitic particles, including the helminth ova and protozoan (oo)cyst, are detected more frequently in wastewater than in other surface waters and are resistant to chlorination or ozonation used commonly in the water and wastewater treatment systems [3,4]. Pathogenic protozoa are major causes of human gastroenteritis transmitted by water and significant challenges of public health worldwide [5]. Several water-borne giardiasis, amoebiasis, cryptosporidiosis, balantidiosis, cyclosporidiosis and microsporidiosis outbreaks have been reported throughout the world [6]. The first outbreak of plant-borne fascioliasis, the biggest in the world, occurred in Iran (Bandar Anzali) in February 1988. Total number of infected inhabitants estimated to be 10,000 [7]. All of trematodes have an indirect life cycle, unlike nematodes, and humans can’t be infected by eating the eggs of these flukes, therefore, the efficiency of wastewater treatment plants in removing eggs can lead to interruption of transmission cycle. Of all the helminth eggs likely to be present in wastewaters, *Ascaris*, *Trichuris* and hookworm are of particular public health concern because of severe socioeconomic consequences of an estimated 39 million DALYs (disability adjusted life year) lost to these infections [8]. Generally, parasite removal in wastewater has been synonymous with the removal of intestinal nematode ova, particularly those of *Ascaris*, *Trichuris* and the hookworms, because they occur commonly and simple purification and microscopic identification methods were available to determine their present. Many of these parasites are zoonotic, thus, can be realized to the importance of various studies based on the removal efficiency of domestic and municipal wastewater treatment plants. Many studies have been conducted to determine the prevalence of these pathogens in surface waters and vegetables in Iran [9-12]. The objectives of the present study were to investigate the occurrence of helminth eggs, protozoan (oo)cysts and pseudoparasitic structures in human and livestock wastewaters, and to evaluate the egg and (oo)cyst removal efficiencies of urban and slaughterhouse wastewater treatment plants by egg and (oo)cyst recoveries in both influent- and final effluent wastewaters.

## Methods

### Study sites and samples

Wastewater samples were collected from three municipal treatment plants and two slaughterhouse treatment plants.

Municipal wastewater treatment plants were located at the west and southwest of Tehran: Shahrak-e Ekbatan (WWTP1), Shahrak-e Gharb (WWTP2) and south Tehran wastewater treatment plant (WWTP3), and recycled water discharged into creek of firoozabad, Sheikh-Fazlollah highway surface water channel and Varamin agricultural lands, respectively.

Domestic wastewater treatment plants were located in one suburb area of Tehran: Meisam-robat-dam (SWWTP4) and Dam-pak (SWWTP5).

The animals slaughtered in these two slaughterhouses were included cattle, sheep and goat, and the plants treated only domestic wastewater and reclaimed water was reused for the irrigation of Shahriār agricultural farm lands.

The main features in each of the five treatment plants are described in Table 1.

**Table 1:** **The main characteristics of the five wastewater treatment plants**

**Wastewater treatment plants**	**Population served**	**Primary treatment**	**Secondary treatment**	**Tertiary treatment and disinfection**	**Biochemical parameter**			Water turbidity*		**Use of treated water**
					**Flow rate (m** ^**3**^ **/s)**	BOD*** **(mg/L)**	TSS**** **(mg/L)**	**influent**	**effluent**	
**WWTP1**	100000	Screening and grit removal	Activated sludge and A2/O*****	Sand filtration followed by chlorination	0.46	<6	<30	High	Moderate	Discharged into creek
**WWTP2**	85000	Screening and grit removal	Conventional activated sludge	Chlorination	0.27	<30	<30	High	Moderate	Discharged to the highway surface water channel
**WWTP3**	2100000	Screening, grit removal and sedimentation	Trickling filter followed by activated sludge	Chlorination and UV radiation	5.2	28	28	High	Moderate	Agriculture irrigation
**SWWTP4**	ND**	Sedimentation	Activated sludge and oxidation with O_2_	Chlorination	ND	ND	ND	High	Moderate	Agriculture irrigation
**SWWTP5**	ND	Sedimentation	Activated sludge and oxidation with O_2_	Chlorination	ND	ND	ND	High	Moderate	Agriculture irrigation

*Low = <1 NTU; moderate = 1–10 NTU; high = >10 NTU; **ND = Not data; ***BOD = Biochemical oxygen demand; ****TSS = Total suspended solids; *****A2/O = Anaerobic/anoxic/oxic.

Grab samples of untreated (5 L each) and treated (10 L each) wastewater were collected at the inlet and outlet points of treatment plants monthly once, from December 2013 to November 2014.

The determination of samples volume was conducted in compliance with Analysis of Wastewater for Use in Agriculture - A Laboratory Manual of Parasitological and Bacteriological Techniques [2].

Water samples were collected in carboys and transported to the intestinal protozoan laboratory of the faculty of public health at the Tehran University of Medical Sciences (TUMS) where they were stored at 4°C until used for the analyses. The samples analyses were completed within 7 days. To reduce the potential for cross-contamination between samples, all sampling carboys used for raw and treated effluent were kept separate.

### Recovery efficiency of *Giardia* cyst concentration procedures

The cyst recovery efficiency of four concentration methods was evaluated and compared to demonstrate acceptable method performance and included (i) modified Bailenger method (MB), as recommended by WHO in “Analysis of wastewater for agricultural use” [2] (ii) scraping and rinsing of membrane method (RM), (iii) acetone-dissolution method (ADM) [13], and (iv) centrifugal-(water-ether) concentration method (CC method) [14].

For each concentration method, six aliquots of 5 litres (untreated and treated) samples were collected. For each concentration procedure, two aliquots of raw and two aliquots of treated samples were used as matrix spikes and were seeded with estimated numbers of *Giardia* cysts.

The cysts used in assays were a gift from Dr. Meamar, School of Medicine, Iran University of Medical Sciences, Tehran, Iran, and were isolated and purified from feces of diarrheal patients by sucrose flotation (specific gravity of 1.18). The number of cysts (~4 × 10^3^) was determined by microscopic examination and the pellet was suspended in one milliliter deionized water.

All of seeded and unseeded samples were sieved through a sries of stainless steel and polyester meshes of 40 (pore size 400-μm), 50 (pore size 297-μm), 200 (pore size 74-μm) and 400 (pore size 37-μm) before filtration to avoid filter blockage by large particles.

The first set of raw (2 seeded and 1 unseeded) and treated (2 seeded and 1 unseeded) samples were transferred to 5000 ml separatory funnel (decanter) (which was designed for this purpose, with two valves built in the bottom of the funnel) and allowed to settle for 24 hours, separately during the consecutive days. The sediments were transferred to 50 ml conical centrifuge tubes through the valves in the bottom of the funnel and centrifuged at 1000 × g for 15 minutes and further processed by modified Bailenger method as described by Mahvi and Kia [10].

The second set of test samples (2 raw seeded, 1 raw unseeded, 2 treated seeded and 1 treated unseeded) were filtered by membrane filters (pore size 0.8-μm, 50-mm diameter; Sartorius, Germany), entrapped particles were achieved by scraping the membrane with a smooth-edged plasticine molder and rinsing with PBS elution fluid (pH 7.4) (containing 0.1% Tween-80 and antifoam agent B) and centrifugation (2100 × *g*, 10 min, 4°C) (RM method).

The third set of test samples (3 raw and 3 untreated) were concentrated by filtration on cellulose-acetate membrane filters (pore size 0.8-μm, 50-mm diameter; Sartorius, Germany) by vacuum pump (model XX 5522050, Millipore). The membrane filters with entrapped particles transferred to a 50 ml conical centrifuge tube, dissolved in acetone and centrifuged (3000 × *g*, 10 min, 4°C). The supernatant fluid was discarded by pipette Pasteur, and the pellet was successively resuspended and centrifuged in 95% ethanol, 70% ethanol, and eluting solution (pH 7.4). The eluting solution consisted of 0.1% (vol/vol) Tween-80 detergent, 0.1% (w/v) sodium dodecyl sulfate, NaCl, KH_2_PO_4_, Na_2_HPO_4_ · 12H_2_O and 0.001% (vol/vol) antifoam agent B (Sigma-Aldrich) (ADM method).

Fourth set of test samples (3 raw and 3 untreated) were centrifuged (2100 × *g*, 15 min, 4°C), and the sediment was concentrated by water-ether concentration procedure following the steps described below.

Microscopic enumeration of cysts was performed in a Thoma counting cell at a magnification of × 400.

The matrix spike recovery was determined from the simple formula as described by Skotarczak [15].

### Parasite particles concentration

The wastewater samples were sieved through a polyester mesh of 40 (400 μm) to remove large particles.

Raw wastewater samples were centrifuged (2100 × *g*, 15 min, 4°C) in a 4 × 500 ml-capacity-swinging-bucket rotor of a refrigerated centrifuge (Beckman, GS-6R Centrifuge).

The supernatant fluids were carefully aspirated by vacuum pump, without disturbing the sediment and about 100 ml of supernatant was left on top of the sediment at the bottom of the canisters (Beckman Aerosolve® Cannisters).

The residues were transferred to 50 ml conical centrifuge tubes and centrifuged as before.

A water-ether concentration procedure was performed with 30 ml deionized water and 9 ml diethyl ether (CC method) [16]. This concentration method was followed by flotation with zinc sulfate (ZnSo_4_ · 7H_2_O) (w/v) (specific gravity 1.364) [17].

The upper layer of the flotation solution was decanted into a container, and this solution was diluted with water to lower the specific gravity of the solution to below that of the protozoan (oo)cysts and helminth eggs, and then the (oo)cysts and eggs were collected in the sediment after centrifugation.

The other hand, treated samples were filtered by cellulose-acetate membrane filter (pore size 0.8-μm, 50-mm diameter; Sartorius, Germany) to retain the particles. Sample elution was achieved by scraping the membranes with a smooth-edged plasticine molder and rinsing with PBS elution fluid (containing 0.1% Tween 80 and 0.001% antifoam agent B). The eluate was collected in a clean glass petri-dish and transferred to a 50-ml centrifuge tube and centrifuged at 2100 × *g* for 10 min.

Microscopic examination and enumeration of (oo)cysts and eggs was performed in a Thoma counting cell at 400× magnification for *Giardia* cysts and other protozoan (oo)cysts and in a McMaster counting cell (weber England) at 100× magnification for helminthic eggs [18].

Protozoan (oo)cysts and helminth eggs were identified by morphometric parasitological criteria including the size, which was measured by a calibrated microscope, and shape of eggs and (oo)cysts at 100×, 400× and 1000× magnifications.

### Data analysis

Results of egg and (oo)cyst quantitation were analyzed with Microsoft Excel (version 2010) and Statistical Package for Social Science version 22 (IBM SPSS Inc., Chicago, IL, USA). Kolmogorov-Smirnov test (test of normality) was realized prior to Student’s *t*-test (Paired *T*-test). Geometric and arithmetic means were also calculated. To evaluate the (oo)cyst and egg removal efficiency of treatment plants, the logarithm of egg and (oo)cyst concentrations were calculated and the difference between the number of eggs and (oo)cysts present in the influent and in the effluent samples was considered. A *p*-value < 0.05 was considered statistically significant.

## Results

### Recovery efficiency of *Giardia* cysts in test samples

**Figure 1:**
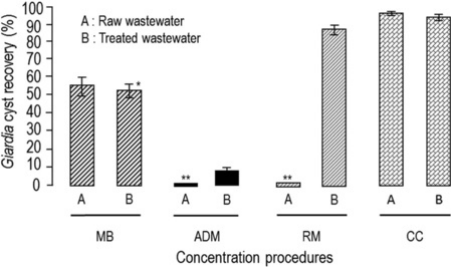
**Comparison of the cyst concentration in raw and treated samples measured by different methods.** Overall mean recoveries are expressed as a percentage. Co-efficient of variance (CV %) was 12, 2.5, lost, 11.8, lost, 1.9, 0.7 and 1.4 in raw and treated samples for MB method, ADM method, RM method and CC method, respectively. *Bars denote SD. **Lost raw samples.

Figure 1 shows the results as the mean percentage recovery and ± SD, and co-efficient of variance.

The microscopic assessment and cyst enumeration results show that CC method has the highest cyst recovery at 96.1% (±0.7) and 95 (±1.4) for raw and treated samples, respectively (Figure 1).

Recoveries of cysts were higher when using RM method than when using ADM method.

The acetone-dissolution method, due to the formation of a hardened pellet (solid robbery pellet) in the bottom of the tube after the centrifugation step that has interfered with the detection of cysts, exhibited cyst recovery of less than expected.

Background solids from the raw test samples contributed to filter blockage for both the scraping and rinsing of membrane method and the acetone-dissolution method, and their results were excluded.

The RM method produced the second highest recovery with 88% (±1.6) cysts isolated for treated samples. The MB method produced the third highest cysts recovery with 55% (±7.0) and 56.4 (±1.4) for raw and treated test samples.

The ADM method exhibited cyst recovery of 10.7% (±1.2) for treated test samples.

Interestingly, a large number of trophozoite forms of protozoa including amoeba, flagellate and cilliate, and a few protozoan cysts, such as *Bodo* spp., *Entamoeba* spp. and *Giardia duodenalis* were found in the supernatant remaining from the modified Bailenger method.

### Frequency and distribution of parasitic particles in raw wastewaters

**Figure 2:**
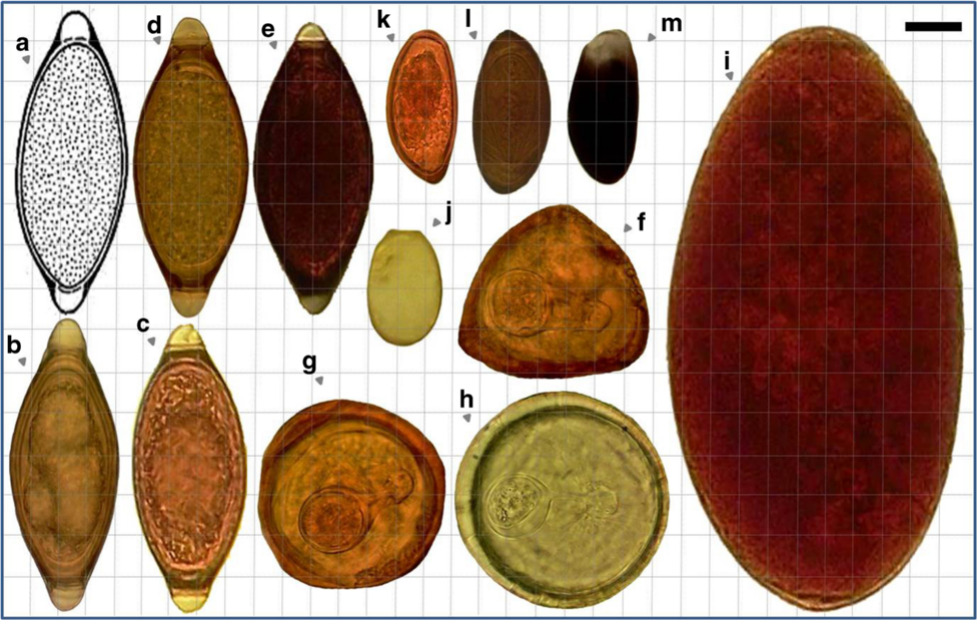
**Relative sizes of some of the parasitic (anthropo-zoonotic) particles found in domestic wastewater treatment plants. (a)**, **(b)**, **(c)** ,**(d)**, **(e)** *Trichuris* spp.; **(f)** *Moniezia expansa* (triangular shape with pyriform apparatus); **(g)**, **(h)** *Moniezia* spp.; **(i)** *Fasciola hepatica*; **(j)** *Eimeria* spp. (damaged oocyst); **(k)**, **(l)** *Dicrocoelium dendriticum*; **(m)** *Dicrocoelium dendriticum* (damaged due to the aeration process) (Photographed under the light microscope at 1000× magnification using by Samsung Mobile, GT-B5512) (Bar showing 20 μm).

**Figure 3:**
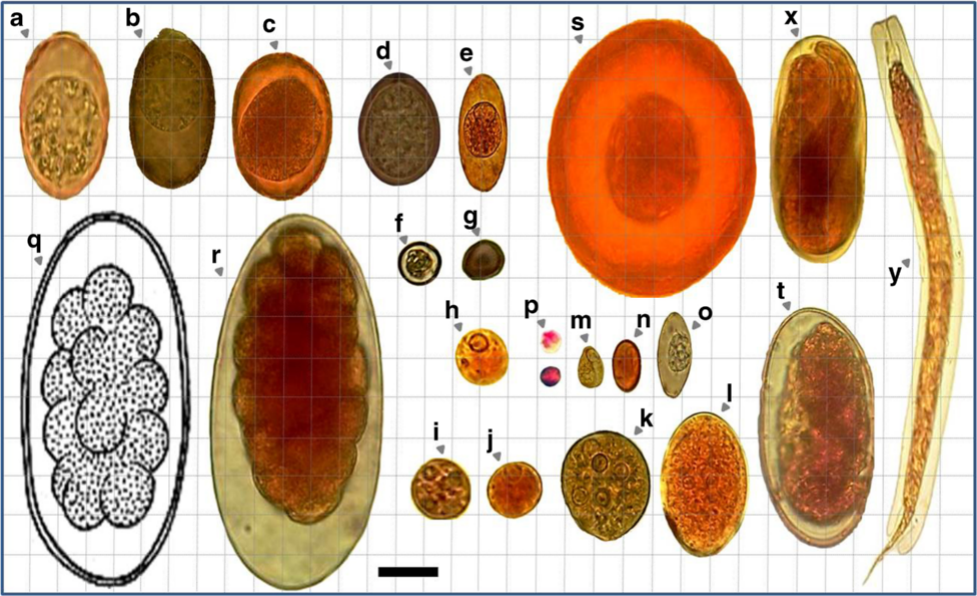
**Relative sizes of some of the parasitic (anthropo-zoonotic) particles found in WWTPs and SWWTPs. a)** *Eimeria* spp.; **(b)** *Eimeria* spp.; **(c)**, **(d)**, **(e)**, **(f)**, **(g)** coccidian spp. (This oocyst is typical of *Eimeria* spp. found in ruminant feces. The micropyle cap would indicate that this specimen came from sheep or goats. Cattle coccidia usually don’t have micropyle caps) (h) *Entamoeba* spp. (*E. ovis*/*debliecki*/*dilimani*/*bovis*); **(i)** *Entamoeba wenyoni*; **(j)**, **(k)**, **(l)** *Entamoeba coli*; **(m)** *Chilomastix mesnili*; **(n)** *Giardia duodenalis*; **o)** *Isospora* spp; **(p)** *Cryptosporidium* spp. (probably *C. andersoni* or *parvum*); **(q)**, **(r)** *Trichostrongylus* spp.; **(s)** *Hymenolepis diminuta*; **(t)** hookworms; **(x)** *Rhabditis* spp. (or probably *Strongyloides*); **(y)** Free living larvae (probably *Rhabditidae*) (Bar showing 20 μm).

**Figure 4:**
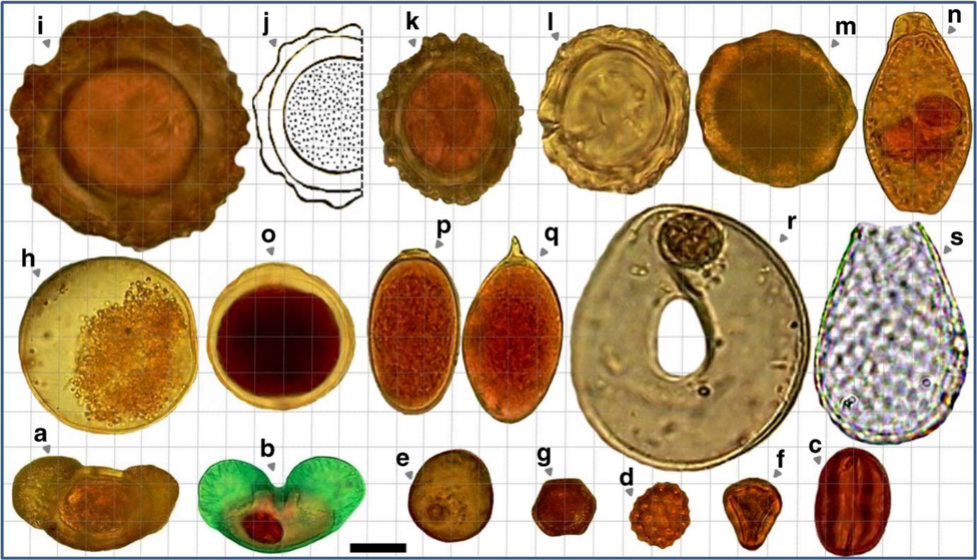
**Relative sizes of some of the pseudo-parasitic particles including pollens, free living protozoa and their fossils found in WWTPs and SWWTPs. (a)**, **(b)** Pollen grain (*Pinus contorta,* Family *Pinaceae*); **(b)** Pollen grain stained with trichrome; **(c)** pollen grain (*Eriogonum crocatum,* Grain Type: Tricolporate)*;* **(d)** *pollen grain* (*Atriplex patula,* Grain Type: Periporate); **(e)** pollen grain; (f) pollen grain (*Eucaliptus globulus,* Myrtaceae Family); **(g)** pollen grain; **(h)** pecan pollen grain; **(i)**, **(k)**, **(l)**, **(m)** *Ascaris* egg-like particles in raw domestic wastewaters; **(j)** *Ascaris lumbricoides*; **(n)** *Epistylis* spp.; **(o)** parasite egg- or oocyst-like particle in municipal raw wastewaters; **(p)**, **(q)** Trematodes egg like particles in raw domestic wastewaters; **(r)** *Arcella discoides*; **(s)** *Euglypha* spp. (Bar showing 20 μm).

Eggs, (oo)cysts and pseudoparasitic structures detected in raw and treated wastewaters are shown in Figures 2, 3 and 4.

(Oo)cysts and eggs were detected in wastewater influents of all plants throughout the year.

In all plants, the highest and the lowest number of (oo)cysts and eggs were found in autumn and summer, respectively.

**Figure 5:**
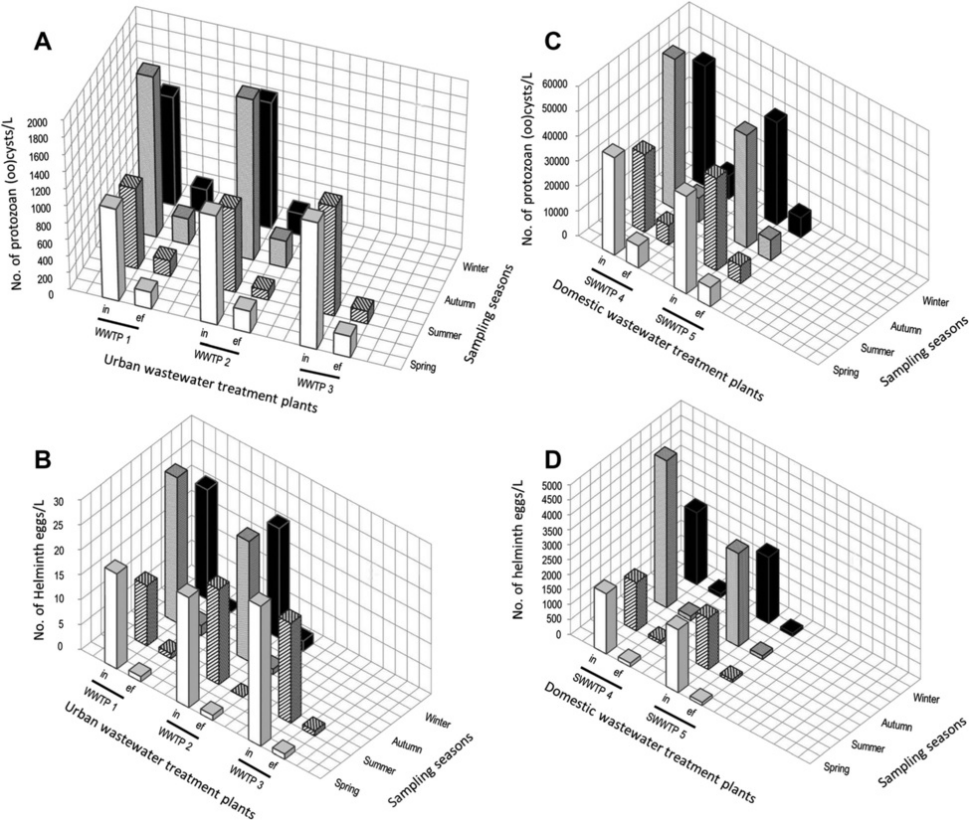
**Number of protozoan (oo)cysts and helminth eggs in wastewater influent samples. (A)** Protozoan (oo)cysts/L of urban wastewaters; **(B)** helminth eggs/L of urban wastewaters; **(C)** protozoan (oo)cysts/L of slaughterhouse wastewaters; **(D)** helminth eggs/L of slaughterhouse wastewaters (in = influent samples; ef = effluent samples).

The estimated number of eggs and (oo)cysts per liter of raw wastewaters from urban treatment plants ranged from 1.2 × 10^1^ to 2.9 × 10^1^ and from 9.6 × 10^2^ to 1.9 × 10^3^, respectively (Figure 5).

**Figure 6:**
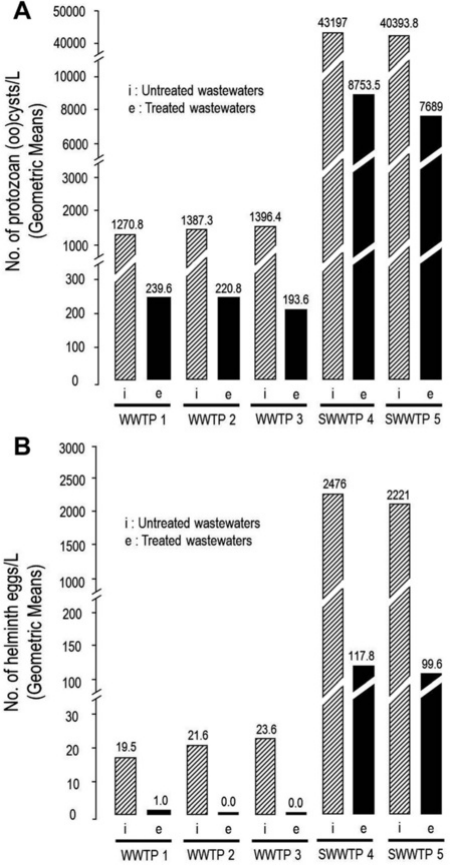
**Geometric mean concentrations of protozoan (oo)cysts** **(A)** **, and helminth eggs** **(B) per liter of raw and treated wastewater samples from urban (WWTPs) and domestic wastewater tratment plants (SWWTPs).**

In the slaughterhouse treatment plants, the estimated number of eggs and (oo)cysts per liter of wastewater influents ranged from 1.6 × 10^3^ to 4.9 × 10^3^ and 31 × 10^3^ to 60 × 10^3^, respectively (Figure 5). Parasite quantification is necessary to evaluate the impact of the wastewater treatment processes on the prevalence of helminth eggs and protozoan (oo)cysts. The geometric mean concentrations of (oo)cysts and eggs per liter of wastewaters are shown in Figure 6.

In livestock wastewaters, eggs belonging to 3 groups of parasitic helminths were identified: the nematodes *Trichuris* spp., *Trichostrongylus* spp., the cestodes *Moniezia expansa* and the trematodes *Fasciola hepatica* and *Dicrocoelum dendriticum*. In urban wastewaters, eggs of 4 groups of helminths were observed: hookworms (*Ancylostoma duodenale* and *Necator americanus*), *Hymenolepis* spp. and *Rhabditis* spp. In these plants, (oo)cysts of 6 groups of parasitic protozoa were found: *Giardia* spp., *Entamoeba* spp., *Cryptosporidium* spp., *Eimeria* spp., *Isospora* spp. and *Chilomastix mesnili* (Figures 2, 3 and 4).

**Figure 7:**
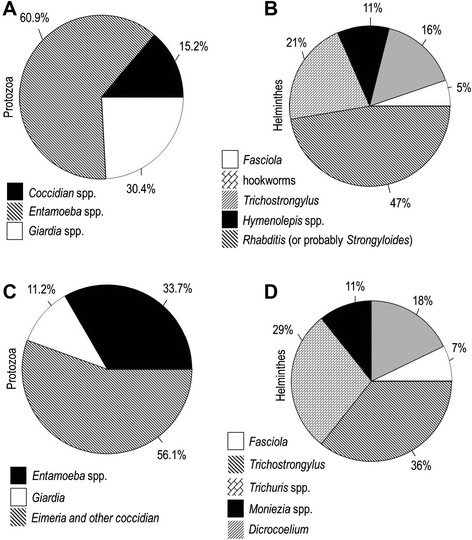
**Cumulative percentages of protozoa (oo)cysts and helminth eggs in raw wastewaters. (A)** and **(B)** municipal wastewaters; **(C)** and **(D)** slaughterhouse wastewaters.

*Entamoeba* spp., the most commonly encountered cysts, were frequently found (60.9%) in urban raw wastewaters (Figure 7).

Sporolated and unsporolated oocysts of *Eimeria* spp. were frequently observed (56.1%) in slaughterhouse wastewater influents (Figure 7).

The highest number of egg in urban and livestock wastewaters was attributed to the nematodes *Rhabditis* (or probably *Strongyloides*) (47%) and *Trichostrongylus* (36%), respectively (Figure 7).

There were a large number of the cystic form particles, with a size of about 40–45 μ, and many pseudo-parasitic structures including protozoan (oo)cyst like particles and helminth egg like particles that we were unable to identify most of them (Figure 4).

We found many type of the small flagellates and amoebas in the raw and treated wastewaters that were rarely encountered.

The types of free-living protozoa and microorganisms in treated wasewater samples including rotifers, water bears, testate amoebas (*Arcella* spp. and *Euglypha* spp.), peritrichs (*Epistylis* spp, *Vorticella* spp.), Carnivorous ciliates (*Acineria uncinata* and *Plagiocampa* rouxi), *Bodo* spp. and *Amoeba* spp. (cysts and trophozoites of unshelled amoebas) were also observed (Figure 4).

### Removal efficiency of treatment plants

The overall average reduction in protozoan (oo)cysts (80.7%) was significant (*P* < 0.05) in all plants (Table 2). A similar reduction was observed in helminth eggs by municipal WWTPs and livestock SWWTPs (*P* < 0.05); however, the difference in reduction was not significant (*P* > 0.110) in WWTP 3 (Table 3).

**Table 2:** **The mean concentrations of protozoan (oo)cyst in raw and treated wastewaters**

**Wastewater treatment plants**	^**†**^ **No. of protozoan (oo)cysts/L samples**		**Mean difference**	**P-value**
	**Influent**	**Effluent**		
**WWTP1**	1315.0 ± 414.2	245.0 ± 59.2	1070.0	0.010
**WWTP2**	1425.0 ± 377.5	237.5 ± 89.6	1187.5	0.004
**WWTP3**	1400.0 ± 141.4	200.0 ± 70.7	1200.0	0.027
**SWWTP4**	44500.0 ± 12449.0	8775.0 ± 694.6	35725.0	0.009
**SWWTP5**	40500.0 ± 3415.7	7700.0 ± 469.0	32800.0	<0.001

^†^Mean ± SD.

**Table 3:** **The mean concentrations of helminth egg in raw and treated wastewaters**

**Wastewater treatment plants**	^**†**^ **No. helminth eggs/L samples**		**Mean difference**	**P-value**
	**Influent**	**Effluent**		
**WWTP1**	20.5 ± 7.0	1.0 ± 0.8	19.5	0.010
**WWTP2**	21.8 ± 2.1	1.0 ± 0.8	20.8	<0.001
**WWTP3**	24.0 ± 5.7	1.0 ± 0.0	23.0	0.110
**SWWTP4**	2725.0 ± 1486.3	118.0 ± 16.7	2607.0	0.038
**SWWTP5**	2275.0 ± 76.8	100.0 ± 9.5	2175.0	0.005

^†^Mean ± SD.

The rate of egg overall removal in plants 1, 2, 3, 4 and 5 was 94.8, 95, 95.5, 95.5 and 95.7%, respectively (Table 4).

**Table 4:** **Overall mean removal efficiency of treatment plants for (oo)cysts and eggs**

**Sampling seasons**	**WWTP1**		**WWTP 2**		**WWTP 3**		**SWWTP 4**		**SWWTP 5**	
	Removal efficiency*		**Removal efficiency**		**Removal efficiency**		**Removal efficiency**		**Removal efficiency**	
	**Cysts and oocysts**	**Eggs**	**Cysts and oocysts**	**Eggs**	**Cysts and oocysts**	**Eggs**	**Cysts and oocysts**	**Eggs**	**Cysts and oocysts**	**Eggs**
**Spring**	81.8	94.7	80	95.4	83.3	92.9	77.4	94	80.5	95.3
**Summer**	80.2	91.7	89	94.7	88.4	95	74.8	94	80.8	94.7
**Autumn**	83.6	93.1	83.1	91.6	ND**	ND	84.3	97.2	81.7	96.3
**Winter**	78.4	95.4	82.6	90.9	ND	ND	81	94.8	80.7	95.4
**Overall mean ± SD** ^**†**^	81 ± 2	93.7 ± 1.6	83.6 ± 3.8	95.5 ± 3.7	85.8 ± 3.6	95.7 ± 0.9	79.3 ± 4.1	95 ± 1.5	80.9 ± 0.5	95.5 ± 0.7

*Removal efficiency calculated as a persentage (%).

**ND, no data.

^†^Mean removal efficiency (%) ± SD.

Results are expressed as the mean percentage removal efficiency and ± SD.

Overall removal efficiencies of protozoa (oo)cysts were also 81, 83.6, 85.8, 79.3 and 80.9% for plansts 1, 2, 3, 4 and 5, respectively (Table 4).

## Discussion

The role played by animal and human wastewaters as sources of parasitic pathogens infecting human is very significant [19].

Many of these intestinal parasites of domestic animals are zoonotic and can be transmitted to humans through ingestion of eggs or (oo)cysts in contamintated water and food (vegetables) and those that do not cause human disease cause severe disease in livestock and have the potential to cause substantial economic losses.

The infected hosts, whether human or animal, shed large numbers of (oo)cysts and eggs via the faeces into the environment, and these parasites are very resistant and may survive in the environment for over a year [20,21].

Moreover, disinfection processes cannot destroy protozoa (oo)cyst and helminths egg, and they have been found in the final effluents of treatment plants [22].

In this study 108 samples were analysed in order to compare the presence of parasitic fauna in untreated and treated wastewaters contaminated by human and livestock feces.

In urban wastewater samples, *Entamoeba coli*, *Entamoeba histolytica*/*dispar*/*moshkovskii*, *Giardia* spp., *Isospora* spp., *Cryptosporidium* spp. and *Chilomastix mesnili* were found.

Our investigation of the municipal treatment plants revealed that *Entamoeba* spp. and *Giardia* spp. were ubiquitous, whereas *Cryptosporidium* spp. were quite rare. Results reported in our study correspond with those from studies published in several countries. Studies carried out in Italia [13,23], China [24], France [25], Malaysia [26], Tunisia [27,28] reported a prominence of *Giardia duodenalis* and *Entamoeba* spp. in municipal raw wastewaters. In a recent study conducted by Kitajima and colleagues [29] in the USA, 24 raw wastewater samples from 2 municipal treatment plants were analyzed for the presence of *Giardia* and *Cryptosporidium*, that the mean concentration of cysts in the influent has been higher than that of oocysts (4800–6400 versus 74–100 (oo)cysts per liter).

In domestic wastewater samples, *Eimeria* spp., *Entamoeba* spp. and *Giardia duodenalis* were frequently descried.

Similar prevalence rates were reported in raw wastewater collected at a slaughterhouse treatment plant in Tabriz, a city in the northwest of Iran, where the species of *E. ahsata*, *E. ovina*, *E. Faurei*, *E. parva*, *E. pallida* and *E. intricata* were identified [30].

In our study, some of the uni-, tetra-, and octanucleated cyst-forming *Entamoeba* species were detected. *Entamoeba wenyoni* in goats is a species of amoebas that has octanucleated cysts. The taxonomic status of these uninucleated *Entamoeba* species over the years has been confusing. They have been identified in cattle, sheep and goats, and have been given separate names, such as *E. bovis* in cattle, *E. ovis* in sheep and *E. debliecki* in goats. However, the various species cannot be distinguished from each other morphologically, and whether they occur in humans or are even genetically distinct remains to be established [31].

The types of helminths ova were observed in this study included gastrointestinal nematodes, cestodes and trematodes.

In the current study, eggs of 5 genus of parasitic helminths were detected in livestock wastewaters: *Trichostrongylus* spp., *Moniezia spp.*, *Fasciola hepatica*, *Trichuris* spp. and *Dicrocoelum dendriticum*.

We also found eggs of 4 genus of parasitic helminths in municipal wastewaters: hookworms (*Ancylostoma duodenale* and *Necator americanus*), *Hymenolepis diminuta* and *Rhabditis* spp. (thin-shell eggs and rhabditoid larvae). These eggs and larvae may be related to the free living forms of *Strongyloides stercoralis*.

It was difficult to distinguish individual species of some parasitic helminths whose eggs and larvae were observed (*Strongyloides* spp, *Rhabditis* spp., *Trichostrongylus* spp. and hookworms), and due to the other objectives pursued in our research project and financial constraints, we did not incubate the above mentioned nematode eggs in order to test their viability.

Results reported in our study correspond with those from studies published in Tehran, Isfahan and Shahrekord cities by our colleagues [10,32].

In our study, helminth ova content in municipal raw wastewater was lower than that reported by Sharafi et al. [33].

Raw wastewater helminth ova contamination found in our study is higher than those of reported for developed countries such as the United State America (1–8 egg per liter) and France (9 egg per liter) and is lower than those of reported for developing countries such as Brazil (166–202 egg per liter), Morocco (840 egg per liter), Jordan (300 egg per liter) [34].

We never found eggs of *Ascaris*, *Taenia saginata*, *Enterobius vermicularis* and *Hymenolepis nana* in untreated human wastewater samples. Most of the eggs were related to aquatic and soil nematodes that are non-pathogenic and the causative agent of spurious infection in humans. In most other studies, these nematode eggs have been reported as Strongyles.

It could stem from the fact that the prevalence of these entric parasites is low in Tehran in the recent years and the fact that eggs of *Enterobius vermicularis* and *Hymenolepis nana* cannot survive in wastewater and are quickly destroyed [35]. *E. vermicularis* and *H. nana* have a direct life cycle and this probably is the secret of their survival.

However, we found some intact eggs of *Hymenolepis diminuta* in urban raw wastewater. This cestode is a common parasite of rats living in sewage drains.

This has led to the suggestion that the enumeration of enteric parasites in wastewaters can be used as an indicator of infection level within a community [36].

Results of parasite removal efficiencies of treatment plants from each of these studies are not strictly comparable since treatment processes were done on different ways. In addition, because of differences in analyses methodologies, the timeframes in which studies were undertaken and how and where samples were collected.

The overall removal efficiency ranged from 79.3 to 85.8% for (oo)cysts and from 94.8 to 95.7% for eggs at the different plants, which is consistent with estimates from other treatment plants that use similar processes [10,33]. Helminth eggs were generally removed more effectively than protozoan (oo)cysts.

Performance for secondary treatment systems for egg removal range from 88 to 97%, whereas removal of (oo)cyst is more variable ranging from 80 to 97% [37].

The highest egg removal efficiency was at SWWTP5 (95.7%) and the highest (oo)cysts removal efficiency was at WWTP3, perhaps as a consequence of employing the trickling filter followed by activated sludge as secondary treatment system.

In the UK Robertson and colleagues reported more variable protozoan removal of 15–99% in activated sludge compared to 5–85% removal in trickling filters but no significant difference in efficacy of protozoan removal was found between the two processes [38].

The overall mean reduction in protozoan (oo)cysts (80.7%) was significant (*P* < 0.05) in municipal WWTPs. A similar reduction was also observed in helminth eggs by municipal WWTPs (*P* < 0.05). The difference in reduction was not significant (*P* > 0.110) in WWTP 3; however, the number of egg in wastewater effluents of the three urban plants was in compliance with the WHO parasitological guideline (<1 nematode per liter) required for irrigation purposes.

The activated sludge process was employed in both SWWTP 1 and SWWTP 2, and their overall mean removal efficiencies were 79.3 ± 4.1 and 80.9 ± 0.5 for (oo)cysts and, 95 ± 1.5 and 95.5 ± 0.7 for eggs. In these plants, the overall mean reduction of (oo)cysts and eggs was significant (*P* < 0.05); however, the number of egg in treated wastewater was not in compliance with the WHO parasitological guidelines.

However, it should be noted that the two slaughterhouse plants were too old and there was no one to give us enough information about the plants.

In these abattoirs, we observed that the rumen contents of slaughtered animals were shipped separately and used for agricultural-land fertilization and hence, it is thought that the use of slaughterhouse wastewater treatment plant is symbolic.

In the present study, in relation to the raw wastewater samples which were analysed by centrifugal-(water-ether) concentration procedure, primary sedimentation resulted in deposition of all particles and secondary sedimentation (the water-ether concentration) resulted in less turbid samples which could be more easily analysed with microscopic method. The use of this procedure significantly improves parasite (oo)cyst and egg purification, particularly for very turbid samples, and is recommended for use in epidemiological studies in which not only (oo)cyst and ova enumeration but also viability assessment are required.

Disadvantage of the membrane filter acetone-dissolution method is the hardening of the pellet containing particles after the centrifugation step and contributes to the overall ≥30 loss of parasitic particles. In addition, the study conducted by Carreno et al. found that the exposure of *Cryptosporidium* oocysts to solutions used for cellulose acetate membrane dissolution filtration reduce their infectivity in HCT-8 cells [39].

The RM method could potentially be used for monitoring parasitic particles in treated wastewater samples and not raw wastewaters in which contain high concentrations of suspended solids.

In the Bailenger method, background solids from the raw wastewater that are immersed in supernatant, not settled solids and particles with a density > 1.05, may have interfered with the precipitation of coccidian oocysts and other protozoan cysts, hence contributing to the overall 30-50% loss.

Dead (oo)cysts are lighter than live (oo)cysts and their settlement is not coincide with the deposition of live (oo)cysts. Therefore, the modified Bailenger method selectively concentrate viable (oo)cysts and more time may be required for settling parasite particles.

We found some of parasite particles consisting of helminth ova and protozoan (oo)cyst (along with trophozoite forms) in municipal and domestic treated wasewater samples.

Also, we found some of free-living protozoa in treated wasewater samples. A complete list of 228 species of protozoa has been reported by [40]. These microorganisms are commonly found in activated sludge and may be mistaken with human parasites.

Disinfection processes in the domestic and municipal wastewater treatment plants cannot destroy protozoa cyst and helminths egg, and they have been found in the final effluents of treatment plants [3,4,41].

Our data provide the first information about the distribution of the zoonotic parasitic particles in wastewater samples from treatment plants and slaughterhouses in Iran. A few studies described the presence of anthroponotic parasites in comparison with zoonotic parasites in faecal samples [25,42,43].

## Conclusions

According to the results the efficiency of removal of nematode eggs and protozoan (oo)cysts in domestic wastewater treatment plants not only is not in compliance with the guidelines for the microbiological quality of treated wastewater used in agriculture but it also is at an alarming rate.

The release of contaminated outflows into surface waters and the use of these contaminated effluents for irrigation activities could increase the risk of human infection with these zoonotic parasites through the consumption of raw fruits and vegetables.

The study results revealed that the efficacy of removal of nematode ova in urban wastewater treatment plants, and not protozoan (oo)cysts, is in compliance with the WHO parasitological guideline (<1 nematode per liter) required for irrigation purposes.

This emphasizes the importance of the protozological control of effluents from urban wastewater treatment plants and the need for regulations that establish the acceptable concentrations of protozoan (oo)cysts based on the use of effluents, i.e., if they should be recycled in the cities for public, for industry, or for irrigation of corps.

However, in many areas, urban wastwater is directly used for the irrigation of corps and animal waste is used for agricultural-land fertilization and hence, these corps are contaminated with some of the zoonotic enteric parasites. Therefore, this is a way for these pathogens to travel further up the food chain, and this could be potential health risk associated with the agriculture application of sludges, manures and raw wastewaters for agricultural irrigation.

## Abbreviations

WWTPs: Wastewater treatment plants
SWWTPs: Slaughterhouse wastewater treatment plants
BOD: Biochemical oxygen demand
TSS: Total suspended solids
A2/O: Anaerobic/anoxic/oxic
NTU: Nephelometric turbidity unit
MB: Modified Bailenger method
RM: Scraping and rinsing of membrane method
ADM: Acetone-dissolution method
CC: Centrifugal-(water-ether) concentration method
